# Ectoparasites and Endoparasites of New Zealand White Rabbits from North West of Iran

**Published:** 2020

**Authors:** Nasser HAJIPOUR, Mohammad ZAVARSHANI

**Affiliations:** 1. Department of Pathobiology, Faculty of Veterinary Medicine, University of Tabriz, Tabriz, Iran; 2. Department of Microbiology, Faculty of Veterinary Medicine, Shahrekord Branch, Islamic Azad University, Shahrekord, Iran

**Keywords:** Ectoparasite, Endoparasite, Parasitic zoonosis, Rabbit

## Abstract

**Background:**

Rabbits contain several parasites that can be harmful to their health as well as human being’s health due to the probability of causing parasitic zoonosis. The present research was designed to study ectoparasites and endoparasites of New Zealand White rabbits in North West of Iran and potential risks of parasitic zoonosis for researchers and owners.

**Methods:**

Totally, 50 rabbits were purchased from rabbit sellers and breeders in suburbs of Urmia and Tabriz between Jul and Dec 2016. The rabbits were assessed for ectoparasites by hair brushing, skin scraping, acetate tape preparation and othic swabs. They were euthanized and inspected for helminths and protozoa infection. Faecal sampling was carried out directly from recti and the oocysts or cysts were isolated using sedimentation and floatation techniques and the sporulated oocyst were identified based on morphological.

**Results:**

The following parasites, with their respective prevalence; Nematoda: *Passalurus ambigus* 54%, *Trichostrongylus retortaeformis* 42%, *Nematodirus leporis* 32%, Cestoda: *Cysticercus pisiformis* 26%, Protozoa: *Eimeria steidae* 44%, *E. magna* 30%, *E. media* 12% and Arthropoda: *Sarcoptes scabiei* 18% and *Cheyletiella parasitivorax* 38%. No significant difference was recorded in infection rate between male and female rabbits.

**Conclusion:**

Both domestic and wild rabbits are a potential source of human parasitic zoonosis, and strict hygienic practices are recommended during and after handling rabbits or in case of exposure to their feces.

## Introduction

Spreading worldwide, rabbits are the small mammals belongs to the family of Leporidae, order Lagomorpha. Until now, 28 races and 77 species of the rabbits have been identified (1). The most common species is New Zealand white rabbit (NZWR).

The rabbits are subjected to various diseases. Among the numerous pathogens that affect the rabbits, parasites play a major role in causing several diseases in rabbit with greater morbidity and mortality leading to economic losses. Some of the parasites are helminths like roundworms, tapeworms, flukes, and the others are ectoparasites like ticks, mites, lice, etc. Ectoparasites are considered as common and important cause of pruritic skin diseases and hypersensitivity disorders in rabbits, as well as some ectoparasites, which are vectors of a wide range of important zoonotic diseases worldwide, such as borreliosis, bartonellosis, ehrlichiosis, rickettsiosis, anaplasmosis, yersiniosis, tularemia (2).

Several studies have been published on the distribution and prevalence of parasites on various breed of rabbits in different parts of the world (3–11). Unfortunately, information regarding prevalence of ectoparasites and gastrointestinal parasites in NZWRs in Iran is scarce (12). Therefore, local and updated information are required since this information gives a great perspective to better understand the epidemiology of intestinal parasitic diseases and the infestation rate of ectoparasites in NZWRs. This information also can be used to take intelligent measures against the disease and is important to prevent the indiscriminate use of anthelmintics that could lead to anthelmintic resistance.

Therefore, this study, for the first time seeks to determine the species and prevalence of ectoparasites and gastrointestinal parasites in NWZRs of North West of Iran, for the first time.

## Materials and Methods

### Sampling

This study was reviewed and approved by the Ethics Committees of Tabriz University, Tabriz, Iran. The present study has been performed since Jul until Dec 2016 based on cross-sectional design. The samples including, 21 (42%) female and 29 (58%) male NZWRs were purchased from two counties where domestic rabbit keeping is common practice in there. These areas included: Urmia (40 from 7 rabbit breeders in Nazlu) and Tabriz (10 from 4 rabbit breeders in Sardroud and Basmenj). The majority of the farms had poor to very poor housing sanitation as characterized by dirty floors, soiled water and feed/feeding equipment, presence of fresh fecal pellets on cage floors. None of the rabbits had received anti-parasitic drugs.

The animals were anesthetized by diethylether and euthanized by intra muscular injection of high doses of the Ketamine 10 % according to the protocol described by Arabali and Hooshyar with some modifications (14).

### Parasitological procedure

#### Ectoparasites

The collected samples were preserved in glass containers with a 70% ethanol solution until identification. Fleas and mites were cleaned by water and immersed in a slightly warm 5% potassium hydroxide (KOH) for 10–15 min, and then samples were transferred to 30% acid alcohol for 5 min. The samples were dehydrated by using series of alcohol from 50% to 100% (absolute) and then cleared by xylene for 5 min. After mounting and drying, the fleas were identified using light microscopic examination, based on the guidelines (13).

#### Gastrointestinal parasites

According to the protocol (14), to collect gastrointestinal parasites, the rabbits were humanely euthanized with slight modifications. Immediately after that, their carcasses were carefully inspected for the presence of protozoa, helminthes. The small intestine of each rabbit was detached from the mesenterium, cut longitudinally, and immersed in phosphate-buffered saline (pH 7.2) for 10 min. The mucosa was scraped with scalpel, thereafter, both the contents of the gut and scraping of the mucosa were washed with tap water in sieve NO. 60 and examined with the unaided eyes as well as under a stereomicroscope (4, 13).

### Fecal examination

To detect the presence of helminthic eggs and protozoan cysts or oocytes, fecal samples collected from rabbits were processed by standard parasitological techniques (direct smear, sedimentation and floatation techniques). The identification of parasitic eggs was done by morphological characters (13). For detection of coccidian oocysts, a part of sample (about 3 gr) was mixed with Zinc sulfate solution (specific gravity- 1.18) and subjected to floatation technique for detection of unsporulated oocysts of coccidia (13). After transferring into 2.5% aqueous potassium dichromate solution (w/v), the collected oocysts were incubated at 25%–28 ºC for 168 h to allow the oocysts to sporulate. The mean sporulation time was assessed by periodically examination of oocysts. The identification of *Eimeria* species has been performed using some biological features such as oocyst size and morphology (curvature, presence or absence of oocyst residuum, conspicuous/inconspicuous micropyle, sporulation time) using the keys previously described by Bowman (4).

### Statistical evaluation

Chi-square test was used for analyzing statistical association between the data findings by using SPSS statistical software (ver. 14, Chicago, IL, USA). Probability of <0.05 was regarded as significant with confidence interval of 95%.

## Results

Among 50 NZWRs used in study 29 (58%) of them were male and 21(42%) were female, forty-two (84%) were infected to at least one of the parasite. Ten species of endoparasite including helminthes and protozoa and two ectoparasites were detected in the rabbits under examination (Table 1). There were three protozoa (56%), one cestode (26%), three nematodes (68%) and two arthropods (52%). *Passalurus ambigus* (54%), *Eimeria stiedae* (44%) and *Trichostrongylus retortaeformis* (42%) were the most common parasites. There was no significant difference in infection rate between male and female NZWRs (*P*>0.05). However, infection rates with exception of *Cheyletiella parasitivorax*, in males was higher than that in females.

**Table 1:** Prevalence of endo and ectoparasites in examined of NZW rabbits (n, %)

***Parasites***	***Sex***		**P*-value***	***Total(n=50)***
	***Female(n=21)***	***Male(n=29)***		
*Nematodirus leporis*	6(28.57)	10(34.48)	0.65	16(32)
*Trichostrongylus retortaeformis*	8(38.09)	13(44.82)	0.63	21(42)
*Passalurus ambigus*	10(47.61)	17(58.62)	0.44	27(54)
*Cysticercus pisiformis*	5(23.80)	8(27.58)	0.76	13(26)
*Eimeria magna*	4(19.04)	11(38.93)	0.15	15(30)
*Eimeria steidae*	8(38.09)	14(48.27)	0.19	22(44)
*Eimeria media*	2(9.52)	4(13.79)	0.18	6(12)
*Sarcoptes scabiei*	3(14.28)	6(20.68)	0.56	9(18)
*Cheyletiella parasitivorax*	8(38.09)	11(37.93)	0.99	19(38)
Infected animals(n,%)	17(80.95)	25(86.20)		42(84)
Non infected(n,%)	4(19.04)	4(13.79)		8(16)

## Discussion

In Iran, NZWRs are mostly kept as pets for children and experimental lab animals due to their low cost, relative size, and perceived ease of care. Rabbits are important as the potential reservoir hosts of a variety of endoparasites and ectoparasites in medical and veterinary point of view and may include various parasites that are transmittable to human (2, 9).

Some of the parasites such as; *S. scabiei* and *C. parasitivorax* found in this study are zoonotic and may cause important public health problems. Therefore, these parasites are potential threats to veterinaries, researchers and public health agencies. In the present study, the overall prevalence of parasitic infection (at least with one of the intestinal helminth species) in NZWRs was 84%, exactly similar to what reported in previous studies which were 79.56% in slaughter rabbits in Poland (11). However, the identified overall prevalence rate in this study was more than that of the earlier researcher, with; 46.67% in wild rabbits of East Azerbaijan Province, Iran (15). The most probable explanation for the high prevalence of parasitic infection in these studies was the poor hygiene, lack of anthelmintic treatment, and favorable climate (16). All of these factors were evident in the present research context.

### Nematoda

*P. ambigus* was the nematode species found in the present survey with the highest prevalence (54%). This is higher than the estimated prevalence previously found in laboratory animals including rabbits in Iran with 40% infection (17), in wild rabbits of East Azerbaijan Province, Iran with 10% (15), in cape hare in Iran with 0.8% (18), in Dutch rabbits in Iran with 6.9% (8). However, the prevalence rate in this study was lower than which found in the earlier studies in wild rabbits (68%) in German by Frank, Kuhn (7). *T. retortaeformis* was the second most abundant species with the prevalence of 42% which was consistent with carried out previous studies in different breeds of rabbits such as; 11.66% in wild rabbits (15). The other nematode species found in our studies was *N. leporis* with a prevalence of 32%, which was more than that found in the earlier studies in Iran with prevalence rate of 13.33% (12).

### Cestoda

From the cestoda order, only *C. pisiformis* was found in NZWRs examined in our studies with prevalence rate of 26% which was more than which found in the earlier studies (5%), (0.4%), (4.74%) (12, 18). The presence of the larval stage of *T. pisiformis* depends on the presence of the definitive carnivore hosts, mostly *Canoidea*. Foxes (*Vulpes vulpes*) are the most common definitive hosts of *T. pisiformis* but dogs and jackals are also infected in Iran (19). The low prevalence of adult *T. pisiformis* and the irregular distribution of this species in this part of Iran was explained by exercising dogs for hunting in rural zones where *T. pisiformis* is found (19).

### Protozoa

In the present study, the prevalence of coccidiosis infection (28%) was consistent with the previous study in Urmia, Iran (67%) (12). However, the prevalence encountered in this study was similar to which reported in previous studies, conducted on different breed of rabbits in other Iranian cities such as; Dutch rabbits in Karaj (21.8%) (8), wild rabbits in Fars Province, Iran (31%) (20) and in other countries, i.e. German (21.2%) (21). The lower prevalence rate of coccidiosis in rabbits from Iran compared to other countries can be attributed to some limiting factors, such as the variations in agro-ecology, meteorology, and environmental conditions prevailing in each region. In addition, the studied rabbits in the countries mentioned are wild, while we studied NZWRs. Humid environment is favorable for oocysts allowing longer life and survival compared to drought and arid conditions. The average annual relative temperature and humidity of Western Azerbaijan Province, North West of Iran is 23 ºC and 38.2%, respectively with annual rainfall of 185 mm. This dry and warm climate is harsh enough to threaten Eimeria life cycle in wild rabbits of this area. Therefore, the observed low rate of coccidiosis in this study is mainly related to dry condition of the region, particularly during recent years._ENREF_58

In our studies, three species of the genus Eimeria were identified, including *E. magna* (30%), *E. steidae* (44%) and *E. media* (12%). The prevalence of 30% for *E. magna* in NZWRs in this study is higher than that reported for wild rabbits from Fars province, Iran (16.9%) (20). However, it was less than those reported by Yakhchali and Tehrani (12) (34.8%). The *E. media* prevalence of 12% found in the current study was similar to that of other studies such as; 8.3% in NZWRs of Urmia, Iran (12) and 14.1% in wild rabbits of Fars province, Iran (20). In the present study, the most common coccidian parasite of rabbits was *E. steidae* with a prevalence rate of 44% which was much more than previous studies done by others such as; (22)(26.86%), (15)(3.33%). These variations may depend on geographical location, the difference in environmental conditions prevailing in each region, the rearing conditions, the number of samples examined, and the season of the year of study (3).

### Arthropoda

*S. scabiei* and *C. parasitivorax*, parasitic zoonoses, were identified in our investigation with prevalence rate of 18% and 38% respectively. The overall prevalence of *S. scabiei* infestation among rabbits was 18%. This finding was far lower than that reported among breeds of Soviet Chinchilla rabbits (57.14%) and NZWRs (28.57%) in previous studies (23). Our results were similar to other studies (22.5%) (5). The poor management of flock by the owner is the main culprit in the high prevalence of mange in the present study causing more susceptibility in the animals (24). The other arthropod separated from studied rabbits was *C. parasitivorax* (38%) identified by in Frank, Kuhn (7) in wild rabbits of German (40%).

## Conclusion

Findings of this study indicated a high prevalence of endo and ectoparasite infections in NZWRs in this particular Iranian context. Some of the identified parasites, known as agents of zoonotic diseases, most probably deteriorate the rabbit’ health, and the zoonotic character of some parasites found in this study must serve as an alert to public health agencies, veterinarians, researchers and pet owners.
